# The effect of primary organic particles on emergency hospital admissions among the elderly in 3 US cities

**DOI:** 10.1186/1476-069X-12-68

**Published:** 2013-08-27

**Authors:** Marianthi-Anna Kioumourtzoglou, Antonella Zanobetti, Joel D Schwartz, Brent A Coull, Francesca Dominici, Helen H Suh

**Affiliations:** 1 Department of Environmental Health, Harvard School of Public Health, Boston, MA, USA; 2 Department of Biostatistics, Harvard School of Public Health, Boston, MA, USA; 3 Department of Health Sciences, Northeastern University, Boston, MA, USA

**Keywords:** Emergency hospital admissions, Fine particles, Medicare, Primary organic particles

## Abstract

**Background:**

Fine particle (PM_2.5_) pollution related to combustion sources has been linked to a variety of adverse health outcomes. Although poorly understood, it is possible that organic carbon (OC) species, particularly those from combustion-related sources, may be partially responsible for the observed toxicity of PM_2.5_. The toxicity of the OC species may be related to their chemical structures; however, few studies have examined the association of OC species with health impacts.

**Methods:**

We categorized 58 primary organic compounds by their chemical properties into 5 groups: n-alkanes, hopanes, cyclohexanes, PAHs and isoalkanes. We examined their impacts on the rate of daily emergency hospital admissions among Medicare recipients in Atlanta, GA and Birmingham, AL (2006–2009), and Dallas, TX (2006–2007). We analyzed data in two stages; we applied a case-crossover analysis to simultaneously estimate effects of individual OC species on cause-specific hospital admissions. In the second stage we estimated the OC chemical group-specific effects, using a multivariate weighted regression.

**Results:**

Exposures to cyclohexanes of six days and longer were significantly and consistently associated with increased rate of hospital admissions for CVD (3.40%, 95%CI = (0.64, 6.24%) for 7-d exposure). Similar increases were found for hospitalizations for ischemic heart disease and myocardial infarction. For respiratory related hospital admissions, associations with OC groups were less consistent, although exposure to iso-/anteiso-alkanes was associated with increased respiratory-related hospitalizations.

**Conclusions:**

Results suggest that week-long exposures to traffic-related, primary organic species are associated with increased rate of total and cause-specific CVD emergency hospital admissions. Associations were significant for cyclohexanes, but not hopanes, suggesting that chemical properties likely play an important role in primary OC toxicity.

## Background

Organic carbon (OC) particles are directly emitted in the atmosphere (primary OC) or formed as a result of photooxidation processes (secondary OC). Both primary and secondary OC are mainly sub-micrometer particles, with a bimodal mass distribution peaking at 0.2 *μ*m and 1 *μ*m [1].

In polluted areas, total OC contributes 10-40% to PM_2.5_ mass [1]. In a study in Los Angeles, total OC contributed 25-45% to PM_2.5_ and 55-75% to ultrafine particle (UFP) mass, across seasons and sites [2]. In general, combustion sources play a major role in the primary OC emissions [3-6], with motor vehicles the main source of many OC compounds, such as hopanes, steranes and cyclohexanes [3,7,8]. For instance, motor vehicle OC comprised approximately 14% of total OC and 5% of PM_2.5_ in Toronto and Vancouver, Canada [9]. Biogenic contributions are also important for the ambient concentrations of specific alkane and PAH species [10,11].

Epidemiologic studies have shown that OC modifies previously reported associations between PM_2.5_ and specific health outcomes [12], suggesting a role for OC in PM_2.5_-related toxicity. Furthermore, given recently published associations between combustion sources-related PM_2.5_ and adverse health impacts [13-15], primary OC compounds and their chemical groups specifically, such as hopanes and traffic-related PAHs, have become the focus of health studies [16,17]. This focus is enhanced by findings from toxicological studies showing impacts of diesel exhaust and UFP on oxidative stress to be mediated by adsorbed organic chemicals rather than the particles themselves [18]. Although these findings suggest that traffic-related organic species play a significant role in the observed PM_2.5_-related toxicity, further research is needed to understand whether and through which species OC may impact human health.

To address this issue, we accessed OC monitoring data from the Aerosol Research and Inhalation Epidemiology Study (ARIES) and Texas ARIES ambient monitoring sites, located in Atlanta, GA, Birmingham, AL, and Dallas, TX. At these sites, a large number (∼120) of individual OC species were measured over multiple years. We grouped the individual OC species by their chemical structures, as these govern their chemical properties [1], to reduce the number of exposure-health comparisons and identify potentially biological relevant chemical properties. We then examined the association between primary OC species as grouped by their chemical structures and cause-specific emergency hospital admissions among Medicare enrollees.

## Methods

Our study was conducted under a protocol approved by the Harvard School of Public Health Human Subjects Committee. Study data did not include individual identifiers and thus consent was not obtained from individuals.

### Data collection

#### Cause-specific hospital admissions data

Data on daily emergency hospital admissions were obtained from billing claims of Medicare enrollees for Atlanta, GA (2006–2009) and Birmingham, AL (2006–2009) and for Dallas, TX (2006–2007). Data for hospitals within Clayton, Cobb, De Kalb, Fulton and Gwinnett counties in Atlanta, Jefferson and Shelby counties in Birmingham and Dallas county in Dallas were included in the analyses. Only admissions that occurred through the emergency department were included, as scheduled admissions are likely not related to short-term air pollution exposures.

Each billing claim contains information on the date of hospitalization, age, residence county and primary diagnoses. Using codes from the *International Classification of Diseases, 9* Revision (ICD-9; Center for Disease Control and Prevention 2008), we considered hospital admissions for all CVD conditions (codes 390–429), for all respiratory outcomes (codes 460–519), and for specific CVD or respiratory conditions: congestive heart failure (CHF; code 428), myocardial infarction (MI; code 410), ischemic heart disease (IHD; codes 410–414), chronic obstructive pulmonary disease (COPD; codes 490–492, 494–496) and pneumonia (codes 480–487). Outcomes were selected based on findings from previous air pollution health studies [12,19,20].

#### Air pollution and meteorologic data

In each city, we obtained daily data for ∼120 non-polar compounds by thermal desorption GC/MS, OC by IMPROVE protocol thermal optical reflectance and PM_2.5_ (measured using 24-hr integrated Federal Reference Methods) measured as part of the ARIES and Texas ARIES studies. The analytical methods are well-accepted and have been previously published [21-23]. Data on temperature and dew point were obtained from Atlanta and Birmingham monitoring sites and the Dallas Fort Worth International Airport.

### Data analysis

All statistical analyses were conducted using the R Statistical Software, version 2.14.1 (Foundation for Statistical Computing, Vienna, Austria).

#### Univariate analyses

We characterized OC particle concentrations using time-series plots, histograms and summary statistics. We further assessed seasonal differences in concentrations, with October–March as the cold period and April–September as the warm period.

OC species were included in further analyses if ≥50% of their observations were above their limit of detection (LOD), ≥75% of the observations were non-missing, and their IQR/median ratio was above 0.30, in all cities. The IQR/median ratio was used instead of the coefficient of variation, given its lower vulnerability to extreme observations. Pollutants with IQR/median ratios ≤0.30 were excluded, as they were not sufficiently variable to allow effect estimation with sufficient power [24].

#### Characterization of primary organic compounds

Primary OC compounds were classified by their chemical structures, as these govern their properties, reactivity and behavior [1]. We categorized OC into six chemical groups: PAHs, n-alkanes, hopanes, steranes, iso-/anteiso-alkanes and cyclohexanes (Additional file 1: Table A-1).

Although not used in the health analyses, the seasonal variability of the organic constituents was also examined, to provide insight in their potential sources. N-alkanes and hopanes were further classified by their sources using well-accepted methods. For alkanes, we estimated city-specific monthly Carbon Preference Index (CPI), as the prevalence of odd to even numbered carbon species, to assess the relative importance of anthropogenic or biogenic sources. For our analyses, CPI values greater than 2 indicated plants and other biogenic sources as the primary n-alkane source, while values near 1 were consistent with anthropogenic sources [25,26]. To identify anthropogenic sources further [3,4,6,10], we also conducted exploratory factor analyses in each city, with the number of factors determined based on (a) identified factors having ≥3 species with a correlation ≥0.30 and (b) a solution that explained ≥90% of the species common variance.

We classified hopane sources in each city using the moretane ratio, with higher ratios indicating greater maturity of the hopanes [26]. This ratio is based on the fact that with increasing thermal maturity, unstable hopanes with hydrogen atoms at the *ββ*-position are transformed to moretanes (*βα*-hopanes) and further to more stable *αβ*-hopanes. Ratios greater than 0.9 indicated that hopanes originated from crude oil, near 0.1 from lignite coal smoke, and 0.4-0.6 from cleaner coals [26].

#### Health models

To assess the effect of primary OC compounds on hospital admissions we used a 2-stage hierarchical regression modeling approach [27], as has been used in studies of dietary exposures and breast cancer [28]. Hierarchical approaches have been widely used in air pollution epidemiology to combine health effects across cities [15,29,30], and more recently across multiple pollutants, such as associations between chemical properties of multiple air pollutants and hospital admissions [24].

In the first stage we fit a case-crossover analysis to the data from all cities. In a case-crossover design, each case acts as their own control, thus eliminating confounding by any personal characteristics that do not change over time [31]. The effect of the exposure on the outcome is then assessed by comparing the distribution of exposures on the days when the case occurred versus the days when the subject did not have the outcome (control days). We modified the time-stratified approach that was proposed by Lumley and Levy [32], employing an ignorable and localizable design [33], choosing control days bidirectionally for subjects within the same city, on the same year and month of the emergency hospital admission, but leaving 3 days between each control day instead of also matching on day of week. By doing so we increased the number of control days, increasing power to detect any effects and by leaving the 3-d buffer we avoided choosing control days too close to the exposure period, which may lead to confounding due to serial correlation [34]. Choosing control days close in time to the admission, furthermore, limits confounding by seasonality and long-term trends [35].

We ran conditional logistic regressions by cause-specific hospital admissions, including simultaneously all eligible primary organic compounds, with their concentrations scaled by their IQR, and adjusted linearly for same day temperature, same day dew point, 1- to 3-day averaged temperature, and day of week. We additionally adjusted for PM_2.5_, as it has been associated with the health outcomes and differentially correlated with the pollutants included in the model as well as with other pollutants not included in the model that could act as confounders [36].

In the second stage we used a multivariate weighted regression model using the coefficient estimates from the first stage as the dependent variables and the variance-covariance matrix of these coefficients as weights. Let *k* denote the number of primary organic compounds in the model and *g* denote the number of pollutant groups, according to their chemical structure:

$$\hat{\beta }=Z\pi +\varepsilon ,$$

where β^k×1 are the coefficient estimates from the first stage, $\underset{k\times g}{Z}$ contains the chemical structure groups (0/1 dummy variables), $\underset{g\times 1}{\pi }$ contains the effect estimates of interest, i.e. the coefficients representing pollutant group effects on the outcome, with each individual coefficient representing the average log rate ratio associated with an IQR increase in that pollutant class, $\underset{k\times 1}{\varepsilon }$ are independent random variables with zero mean and pre-specified variance *τ*^2^, with *k* = 58 and *g* = 5. Since the second stage includes all chemical structure groups in which the organic compounds we used belong, the existence of any residual associations seems unlikely and we therefore set the *τ*^2^ to a modest value, i.e. *τ*^2^ = 2.6 × 10^−5^. Because 100 × [exp(2 × 1.96×*τ*) − 1] ≈ 2, if *τ* = 0.0051, our selected value for *τ*^2^ corresponds to expectations that 95% of the % changes would fall within a 2-fold range.

We examined associations between cause-specific emergency hospital admissions and weekly (7-d) exposures to primary organic compounds, with exposure windows chosen based on previous literature [17]. We also examined same day exposures and moving averages of 2-, 4-, and 6-days. We call statistically significant effects those whose 95% confidence intervals do not include 0.

We examined potential multicollinearity among the groups using the eigenvalues of the variance-covariance matrix of the second stage effects. Based on this examination, sterane effects were found to be highly correlated with hopane (r = -0.69), n-alkane (r = 0.78) and cyclohexane (r = -0.88) effects and were thus excluded from further analysis.

#### Sensitivity analyses

We conducted a series of sensitivity analyses to assess the robustness of our results.

##### Limit of detection

We ran two-stage models for total CVD and respiratory emergency hospital admissions, including species with at least 75% of observations above the limit of detection, to examine sensitivity of our results to LOD exclusion criteria. For this analysis a total of 40 species were included, as compared to 58 in the main analysis: 14 n-alkanes, 2 PAHs, 4 iso-/anteiso-alkanes, with the same number of cyclohexanes (2) and hopanes (18) as in the main analysis.

##### Effect estimate stability

We assessed the stability of the effect estimates (as % change in group effects and width of confidence intervals) by excluding individual chemical groups from the analysis one-by-one and assessing change in the results.

We also assessed the sensitivity of our results to the inclusion of PM_2.5_ in the health models, by repeating analyses omitting PM_2.5_.

##### Sensitivity of our results to the choice of the *τ*^2^ value

We assessed the dependence of our results for total CVD and total respiratory admissions on our pre-specified value of *τ*^2^, by exploring different values for *τ*^2^. Specifically, we also examined *τ*^2^ = 0, allowing the variability of the second stage effects to only depend on the variance-covariance matrix of the first stage coefficients, and also *τ*^2^ = 0.0001, corresponding to expectations that 95% of the % changes would fall within a 4-fold range.

##### Temperature effects

Given observed associations between extreme temperatures and adverse health [37,38], we examined whether our findings were affected by extreme temperatures. We did so by excluding the 99^th^ and 1^st^ percentiles of daily temperatures from our health models.

## Results

City-specific summary statistics for the primary organic compounds used in our analyses are presented in Additional file 1: Tables A-2, A-3 and A-4. Primary organic compounds in our analyses accounted for 14.5%, 17.6%, and 10.8% of the total measured OC (both primary and secondary) mass concentration in Atlanta, Birmingham, and Dallas, respectively. City-specific summary statistics for total CVD and respiratory, and cause-specific, hospitalizations are presented in Additional file 1: Table A-5.

### Characterization of primary organic particle concentrations

#### n-Alkanes

Of the primary organic compounds, n-alkanes generally had the highest concentrations in all three cities. In Atlanta and Dallas, concentrations of n-alkanes were higher during the colder period (October–March) as compared to warmer months. Similar seasonal patterns were found in Birmingham for all primary organic compounds except those with 24-29 carbons (i.e. *C*_24_– *C*_29_), for which no seasonal variation was found. For all n-alkanes, the highest and most variable concentrations were observed in Birmingham, while the lowest and least variable were found in Dallas.

**Figure 1:**
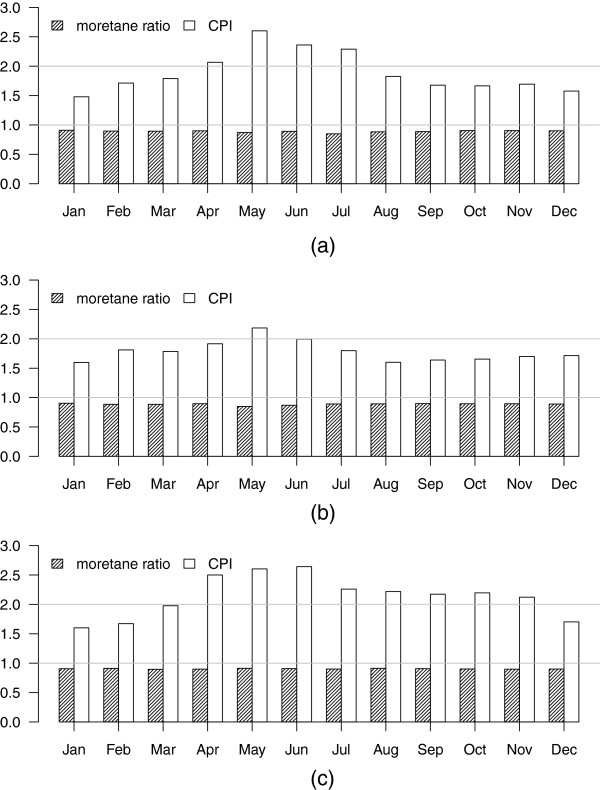
Monthly CPI and moretane ratios for (a) Atlanta, (b) Birmingham and (c) Dallas.

In all three cities, the CPI ranged approximately between 1.5 to 2.6 (Figure 1), indicating mixed anthropogenic and biogenic sources of n-alkanes. CPI values varied seasonally, with peaks in May and June, suggesting increased plant contributions during these months. Correspondingly, we found three sources of n-alkanes using factor analyses, consistent with vehicular emissions, tire debris and plant contributions (Additional file 1: Table A-6).

#### Iso-/anteiso-alkanes

Concentrations of iso- (2-methyl) and anteiso- (3-methyl) alkanes, which are considered as environmental tobacco smoke (ETS) markers in urban air [39], were highest during colder months in all three cities. Concentrations were highest in Atlanta and lowest in Dallas.

#### Cyclohexanes

Concentrations of cyclohexanes were generally low, with only two cyclohexanes, heptadecylcyclohexane and nonadecylcyclohexane, having more than 50% of their observations above their LODs in each city. Both cyclohexanes, which are emitted from gasoline-powered motor vehicles [8] and diesel fuel [7], had higher cold-period concentrations in all cities. Concentrations of heptadecylcyclohexane were similar across all cities, while of nonadecylcyclohexane were highest in Birmingham and lowest in Dallas.

#### Hopanes

Hopane concentrations were also higher in each city during the colder months. The highest concentrations of hopanes were observed in Birmingham and the lowest in Dallas. Monthly moretane ratios in all three cities were greater than 0.85 in Atlanta and Birmingham and than 0.90 in Dallas (Figure 1). Ratios near one, together with the higher cold-period concentrations, are consistent with motor vehicles being the primary hopane source.

#### PAHs

14 PAHs had more than 50% of their observations above their corresponding LOD in the three cities. Cold-period concentrations of these PAHs were higher than those during warmer months. All PAH concentrations were highest and more variable in Birmingham and lowest in Dallas, except from retene concentrations that were similar in both Birmingham and Atlanta and lower in Dallas.

### Relationship of primary OC compounds with emergency hospital admissions

#### Emergency hospital admissions for total CVD

The associations between CVD admissions and PM_2.5_, total OC (both primary and secondary) and EC are shown in Additional file 1: Table A-7.

**Figure 2:**
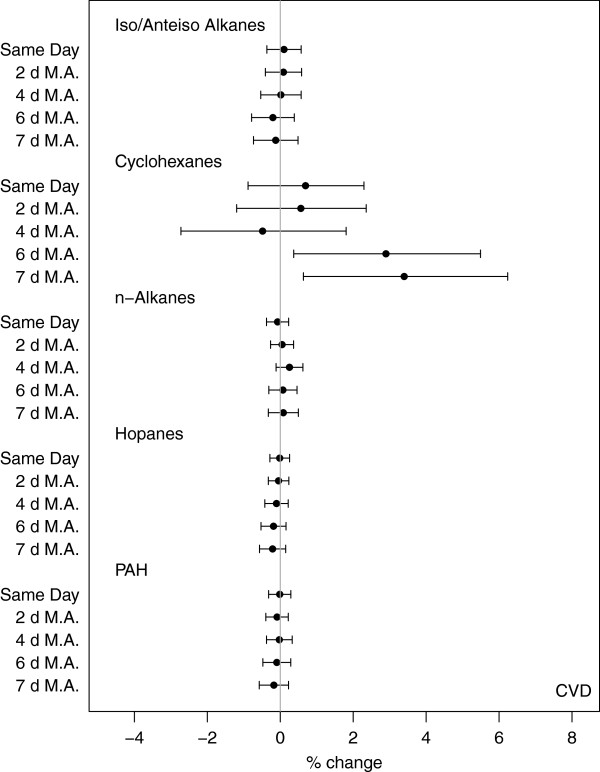
Percent change in total CVD hospital admissions per IQR increase in pollutant group for all exposure windows – same day exposures and 2-, 4-, 6-, and 7-d moving averages (M.A.).

Figure 2 shows associations between chemical property groups and total CVD-related emergency hospital admissions. Overall, significant associations were observed for longer moving averages and more specifically for the 6-, and 7-d moving averaged exposures (Additional file 1: Table A-8).

Cyclohexanes were overall positively associated with CVD admissions, with increasing effects observed with increasing exposure durations. An IQR increase in the 7-d exposure to cyclohexanes was associated with a 3.40% (0.64, 6.24%) increase in the rate of CVD admissions. Increased rate of CVD admissions were also significantly associated with 6-d (2.90% (0.37, 5.49%)) moving averaged exposures. There was no evidence of an association between n-alkanes, iso-/anteiso-alkanes, hopanes and PAH and CVD-related hospital admissions; we saw null associations between all other groups and rate of hospitalizations for all exposure windows.

#### Emergency hospital admissions for total respiratory outcomes

The associations between respiratory admissions and PM_2.5_, total OC and EC are shown in Additional file 1: Table A-7.

**Figure 3:**
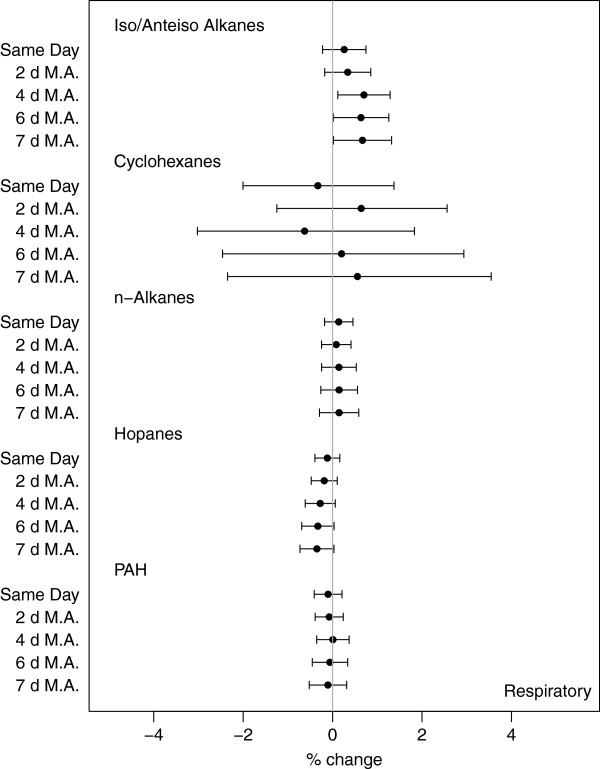
Percent change in total respiratory hospital admissions per IQR increase in pollutant group for all exposure windows – same day exposures and 2-, 4-, 6-, and 7-d moving averages (M.A.).

The associations between exposures to primary OC particle groups and rate of respiratory-related emergency hospital admissions are presented in Figure 3 for all exposure windows. As with cardiovascular associations, we saw increasing effect estimates at longer moving averaged exposures (Additional file 1: Table A-8). We observed increased rate of respiratory hospitalizations after 4-d exposures to iso-/anteiso-alkanes (0.70% (0.12, 1.29%)), with similar increases for 6- and 7-d exposures (0.67% (0.02, 1.32%) for weekly exposures). Overall, there was consistent evidence that the iso-/anteiso-alkanes are positively associated with respiratory hospital admissions for all exposure windows, with increasing effects for longer exposures. Negative, albeit not significant, associations were found for hopanes, with higher effects observed for 7-d (-0.35% (-0.73, 0.03%)) exposures.

#### Cause-specific admissions

**Figure 4:**
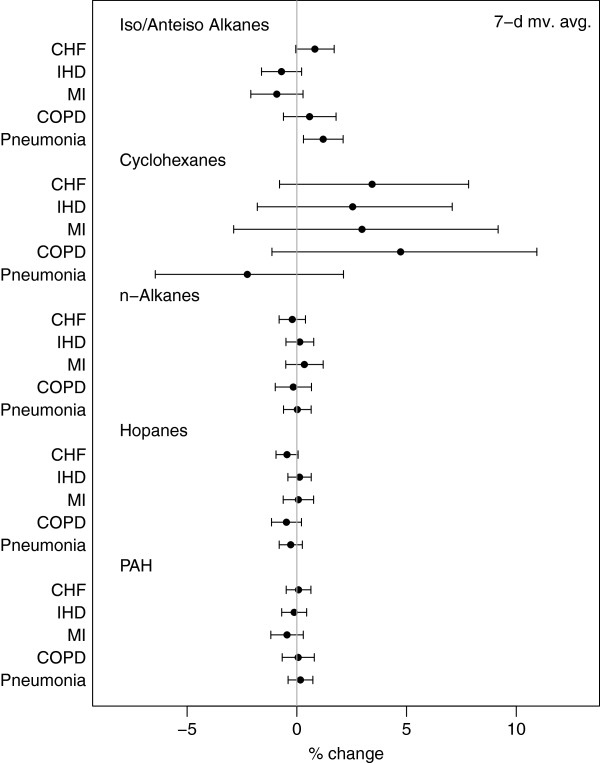
Percent change in cause specific hospital admissions per IQR increase in pollutant group for weekly exposures.

The associations between primary organic compounds and hospitalizations for the specific cardiovascular-related causes IHD, MI, and CHF are shown in Figure 4 and in Additional file 1: Figure A-1 and Additional file 1: Table A-8.

Results for IHD and MI were similar to those for total CVD. For both outcomes, we saw positive associations between increased rate of hospital admissions and weekly exposures to cyclohexanes; 6-d exposures were associated with a 3.65% (-0.36, 7.83%) rate of IHD hospital admissions and 3.21% (-2.10, 8.81%) rate for MI admissions. In addition, we observed a negative, albeit insignificant, association between rate of IHD and MI hospitalizations and exposures to iso-/anteiso-alkanes. For CHF, we found associations between same day cyclohexane exposures and increased rate of hospitalizations (1.58% (-0.60, 3.80%)). Similar increases were also observed for 6- and 7-d exposures. Association with CHF admissions were not detected for other chemical groups, other than a negative association with weekly hopane exposures (-0.45% (-0.95, 0.06%)).

The results for weekly exposures and cause specific respiratory admissions are presented in Figure 4, Additional file 1: Table A-8, and Additional file 1: Figure A-2.

We saw consistent associations between cyclohexane exposures and COPD admissions, which increased with longer exposure windows (4.73% (-1.13, 10.94%) for 7-d exposures). In contrast, we observed consistently negative, mostly insignificant, associations between rate of pneumonia and exposure to cyclohexanes, with stronger effects for 4-d moving averages (-3.65% (-7.04, -0.13%)).

Similarly to total respiratory admissions, we observed increased rate of pneumonia hospitalizations and increased exposures to iso-/anteiso-alkanes for all exposure windows (1.07% (0.22, 1.93%) for 6-d and 1.20% (0.30, 2.11%) for 7-d exposures). Moreover, we found an increased rate of pneumonia admissions after 4-d exposure to n-alkanes (0.38% (-0.15, 0.92%)). We did not detect any association between either hopanes or PAHs and rate for cause-specific respiratory-related hospital admissions.

### Sensitivity analyses

#### Limit of detection

Analyses including species with at least 75% of the observations above the LOD showed similar results for CVD admissions to those from the main analyses, except for those for PAHs, for which associations were now negative, although not significant (Additional file 1: Figure A-3). Furthermore, confidence intervals for PAH were substantially wider, reflecting the inclusion of only two species instead of 14.

For total respiratory admissions, the effects of cyclohexanes and hopanes were similar as in the main analysis. The associations with PAHs were now negative and with n-alkanes positive, albeit still not significant. Finally, we observed positive, but insignificant, associations with iso-/anteiso-alkanes, for all exposure windows (Additional file 1: Figure A-3), in contrast to the significant positive results in the main analysis.

#### Effect estimate stability

When steranes were included in the analysis, effect estimates were similar, but with wider confidence intervals due to collinearity. When chemical groups were excluded from our analyses either individually or in groups, our results were stable, indicating that no multicollinearity among the chemical groups was present after exclusion of steranes. The effect estimates and their variability also did not change when we omitted PM_2.5_ from the health models.

#### Sensitivity of our results to the choice of the *τ*^2^ value

Overall, we saw no differences in the second stage effect estimates for different *τ*^2^ values. As, expected, with increasing values for *τ*^2^ we observed wider confidence intervals. For total CVD admissions the increase of the widths of the confidence intervals was small and the effect estimates remained stable (Additional file 1: Figure A-4). In contrast, the increase of the widths of the confidence intervals was larger for total respiratory hospital admissions, but also the effect estimates did not appear as stable (Additional file 1: Figure A-5).

#### Temperature effects

Our results did not change when we excluded the 1^st^ and 99^th^ percentiles of daily temperatures from our analyses (results not shown).

## Discussion

We conducted a large, multi-year, multi-city, exploratory study to investigate exposures to primary OC compounds and the association between their chemical classes and emergency hospital admissions among an elderly population. Using a hierarchical two-stage modeling approach, we found consistent associations between grouped primary OC compounds and cause-specific hospital admissions. Our most consistent associations were found between cyclohexane and iso-/anteiso-alkane exposures and CVD- and respiratory-related admissions, respectively. Since the primary sources of cyclohexanes are motor vehicles, our results provide support for the growing literature showing motor vehicle-associated adverse CVD effects [14,40], as main sources of cyclohexanes are motor vehicles. At the same time, however, we saw either null or negative associations for hopanes, also markers of motor vehicle pollution, suggesting that the OC chemical structure might be important to toxicity. Moreover, our findings of positive associations between total respiratory and pneumonia admissions and iso-alkanes, a group that has not otherwise been examined in epidemiologic studies, are consistent with previous studies that found community-acquired pneumonia to be associated with inhalation of tobacco smoke [41]. Given the lack of studies examining the relation of these OC classes and health, our findings should be viewed as preliminary and should be validated in additional studies.

Despite this, our findings of adverse cyclohexane impacts are supported by toxicologic studies, which have linked traffic-related particles with numerous adverse outcomes. There is evidence that the redox chemistry of organic particles plays an important role in the biological effects of ambient PM_2.5_[42]. Nel et al. [43], for instance, found organic compounds from diesel exhaust to induce reactive oxygen species (ROS) in macrophages and bronchial epithelial cells, as well as apoptosis and necrosis in the epithelial cells in the bronchial region. Furthermore, Li et al. [16] showed that expression of heme oxygenase-1, a marker for oxidative stress, is directly correlated with high organic carbon content of UFPs. Although there have been no studies examining the toxicological effects of cyclohexanes, it is possible that cyclohexanes may lead to adverse health effects through similar biological processes, since they, like PAHs, can also be transformed by photochemical reactions into more polar products [44,45]. If so, it is possible that cyclohexanes are acting as surrogates of atmospheric processed, oxidized compounds.

Few studies have examined health impacts from specific organic carbon chemical classes, with these studies focusing on PAHs and hopanes. In a Los Angeles cohort of 60 elderly subjects with coronary artery disease, Delfino et al. [17] found significant associations between intermediate markers of inflammation and weekly exposures to PAHs and hopanes. Kraus et al. [46] reported significant associations between exposures to hopanes and PAH and shortness of breath and between PAH and increased C-reactive protein in a cohort of MI survivors in Germany. These findings differ from those in our study, as we found null associations between PAHs and hopanes and increased hospital admissions. The contrasting findings likely result from several key differences in our studies. The earlier studies used different study designs and focused on different health outcomes and examined potentially more susceptible populations, for which primary OC-mediated health risks may differ. In addition, the earlier studies measured a larger number of traffic-related PAHs, e.g. 24 [17], which may provide more power to detect associations.

Support for our findings is provided by the consistent trends found across exposure windows, with larger associations for both cyclohexanes and iso-/anteiso-alkanes found at exposure windows of 6-days and longer. These longer exposure windows may reflect biological processes, such as inflammation, which have been shown to occur over week-long time periods [47]. Alternately or in addition, significance at longer exposure windows may reflect increased classical error associated with shorter averaging times [48]. The wider CIs at longer moving averages could also reflect loss of power; as the averaging period increases the variability in pollutant concentrations decreases.

The robustness of our findings is further supported by consistent findings from our sensitivity analyses. When we restricted our inclusion criteria to species with ≥75% observations above the LOD, results for cyclohexanes and hopanes were similar, while results for PAHs and n-alkanes became negative and positive, respectively, and associations between iso-alkanes and total respiratory admissions became insignificant. The exclusion of species with lower concentrations could result in either decreased noise, as in the case of the PAHs and n-alkanes, or decreased power, as in the case of the iso-alkanes, depending on how conservative the LOD is and the fraction of observations below the LOD. It is also likely, nonetheless, that the exclusion of several species will make the results non-interpretable as a group. For example, in the sensitivity analysis, we only included one softwood and biomass combustion marker (retene) and a general combustion marker (fluoranthene) in the PAH group. It is likely that these two components are not representative of the PAH group, since more condensed PAHs with a wider range of molecular weights and sources were excluded.

Our sensitivity analysis on the choice of the *τ*^2^ provides further insight in our results. For total CVD admissions, our results were stable and did not vary with *τ*^2^. In contrast, our results for total respiratory hospitalizations were less stable, as evidenced by the second stage effect estimates and their confidence interval widths that differed widely across different values of *τ*^2^.

Our study has several limitations. First, ambient measurements were made at a single monitoring site in each city. All pollutants measured are emitted from local sources and thus expected to be spatially heterogeneous. The resulting exposure error, nonetheless, is likely to bias our results toward the null [49]. There might still remain some residual confounding by time-varying variables; this is unlikely, however, given our use of a case-crossover design [33,34]. Furthermore, many species did not meet our inclusion criteria and thus were not included in the analyses. Additionally, we only included particulate OC species. As a result, chemical groups were incompletely represented, as the chemical groups also include species in the gaseous phase. It is likely, however, that gases and particles exert different toxicities. Also, to increase power, we selected our controls leaving 3 days between control days, resulting in overlapping exposure periods between the cases and controls for the longer moving averages examined, and thus potential bias toward the null [33]. Our results are limited by the number of compounds measured and included in the analysis and should be, therefore, interpreted in light of these limitations.

To our knowledge, this has been the first study to examine association between primary organic compounds and hospital admissions. Overall, our results suggest that primary OC compounds, such as cyclohexanes, alkanes and iso-/anteiso-alkanes, may play an important role in PM_2.5_ toxicity and lend some support for previous findings of associations between hospitalizations and mobile sources. Furthermore, our findings add to the current research, provide insight for pollutant groups that have not previously been studied and guidance for the design of future epidemiologic and toxicologic studies.

## Conclusions

Our findings suggest that mobile source effects on CVD hospitalizations could be linked to cyclohexanes, a pollutant group that has not been studied before in any epidemiological or toxicological setting. We saw consistent associations between exposure to particulate cyclohexanes and increased rate of total and cause-specific CVD emergency hospital admissions. This approach should be replicated in more cities and using more compounds in each group to assess the robustness of our findings.

## Abbreviations

CHF: Congestive heart failure; COPD: Chronic obstructive pulmonary disease; CVD: Cardiovascular disease; EC: Elemental carbon; ETS: Environmental tobacco smoke; IHD: Ischemic heart disease; MI: Myocardial infarction; OC: Organic carbon; PAH: Polycyclic aromatic hydrocarbon.

## Competing interests

The authors declare that they have no competing interests.

## Authors’ contributions

MAK was responsible for design, conduct, analysis, interpretation of data and writing the manuscript. AZ assisted with the statistical analyses. JS made contributions to design, analysis and interpretation. BC participated as statistician and in the compilation of the exposure data and interpretation of the results. FD also participated as statistician and in the compilation of the health data and HS made contributions to conception, design, compilation, analysis of data and drafting the manuscript. All authors read and approved the final manuscript.

## Supplementary Material

Additional file 1 Appendix. Click here for file
