# Supplementary material for: The effect of primary organic particles on emergency hospital admissions among the elderly in 3 US cities
Source: Environ Health. 2013 Aug 27;12:68. doi: 10.1186/1476-069X-12-68 (PMC3765898; doi:10.1186/1476-069X-12-68)
Supplement: Additional file 1 — Appendix. [file 1476-069X-12-68-S1.pdf]

Appendix

The Effect of Primary Organic Particles on Emergency Hospital Admissions among the Elderly in 3 US Cities

Marianthi-Anna Kioumourtzoglou, Antonella Zanobetti, Joel Schwartz, Brent A. Coull, Francesca Dominici, Helen Suh

Contents

List of Tables

A-1 Primary organic particles by chemical structure. The species used in our primary analyses are in **bold**.
A-2 Pollutant summary statistics, Atlanta. Concentrations expressed in ng/m^3, except from OC (μg/m^3) .
A-3 Pollutant summary statistics, Birmingham. Concentrations expressed in ng/m^3, except from OC (μg/m^3)
A-4 Pollutant summary statistics, Dallas. Concentrations expressed in ng/m^3, except from OC (μg/m^3) . .
A-5 n-Alkane factor analysis results, by city. Correlations < 0.40 are not shown for better identification of the factor loadings. . . . .
A-6 Cause-specific daily emergency hospital admissions, by city. . . . .
A-7 Percent change (%) in total CVD and respiratory hospital admissions per 10 μg/m^3 of $PM_{2.5}$ and 1 μg/m^3 of OC and EC increase for 2- and 7-d averaged exposures. . . . .
A-8 Percent change (%) in cause specific hospital admissions per IQR increase in pollutant group for 6- and 7-d averaged exposures. . . . .

List of Figures

A-1 Percent change in (a) CHF, (b) IHD and (c) MI hospital admissions per IQR increase in pollutant group for all exposure windows – same day exposures and 2-, 4-, 6- and 7-d moving averages (M.A.). . . . .
A-2 Percent change in (a) COPD and (b) pneumonia hospital admissions per IQR increase in pollutant group for all exposure windows – same day exposures and 2-, 4-, 6- and 7-d moving averages (M.A.).
A-3 Percent change in total (a) CVD and (b) respiratory hospital admissions per IQR increase in pollutant group for all exposure windows, when species with at least 75% of available observations above the LOD were included in the analysis. . . . .
A-4 Sensitivity analyses on the choice of τ^2: Percent change in total CVD admissions for (a) $\tau^2 = 0$, (b) $\tau^2 = 0.0051$ (main analysis) and (c) $\tau^2 = 0.0010$ per IQR increase in pollutant group for all exposure windows. . . . .
A-5 Sensitivity analyses on the choice of τ^2: Percent change in total respiratory admissions for (a) $\tau^2 = 0$, (b) $\tau^2 = 0.0051$ (main analysis) and (c) $\tau^2 = 0.0010$ per IQR increase in pollutant group for all exposure windows. . . . .

Table A-1: Primary organic particles by chemical structure. The species used in our primary analyses are in **bold**.

Pollutant Category	Primary Organic Particles
n-Alkanes	n-pentadecane, n-hexadecane, n-heptadecane, n-octadecane, n-nonadecane, n-icosane, **n-heneicosane**, **n-docosane**, **n-tricosane**, **n-tetracosane**, **n-pentacosane**, **n-hexacosane**, **n-heptacosane**, **n-octacosane**, **n-nonacosane**, **n-triacontane**, **n-hentriacontane**, **n-dotriacontane**, **n-tritriacontane**, **n-tetratriacontane**, **n-pentatriacontane**, **n-hexatriacontane**, **n-heptatriacontane**, n-octatriacontane, n-nonatriacontane
Iso/anteiso-alkanes	**anteiso-nonacosane**, **anteiso-triacontane**, **anteiso-hentriacontane**, **anteiso-dotriacontane**, anteiso-tritriacontane, **iso-nonacosane**, **iso-triacontane**, **iso-hentriacontane**, iso-dotriacontane, iso-tritriacontane, iso-tetratriacontane
Cyclohexanes	octylcyclohexane, decylcyclohexane, tridecylcyclohexane , **heptadecylcyclohexane**, **non-adecylcyclohexane**,
Hopanes	**$\alpha\alpha$-$\beta\alpha$-norhopane**, **$\alpha\alpha$-hopane**, **$\alpha\beta$-hopane**, **$\alpha\beta$-norhopane**, **$\alpha\beta$R-bishomohopane**, **$\alpha\beta$R-homohopane**, **$\alpha\beta$S-bishomohopane**, **$\alpha\beta$S-homohopane**, **$\beta\alpha$-hopane**, **22,29,30-norhopane**, **22,29,30-trisnorneophopane**, **22,29,30-trisnorphopane**, **22R-pentashomohopane**, **22R-tetrashomohopane**, **22R-trishomohopane**, **22S-pentashomohopane**, **22S-tretrahomohopane**, **22S-trishomohopane**
Steranes	$\alpha\alpha\alpha$20R-Cholestane, $\alpha\alpha\alpha$20R24R-Ethylcholestane, $\alpha\alpha\alpha$20R24R-Methylcholestane, $\alpha\alpha\alpha$20S-Cholestane, $\alpha\alpha\alpha$20S24RS-Ethylcholestane, $\alpha\alpha\alpha$20S24S-Methylcholestane, $\alpha\beta\beta$20R-Cholestane, $\alpha\beta\beta$20R24R-Ethylcholestane, $\alpha\beta\beta$20R24S-Methylcholestane, $\alpha\beta\beta$20s-Cholestane, $\alpha\beta\beta$20S24R-Ethylcholestane, $\alpha\beta\beta$20S24S-Methylcholestane
PAH	acenaphthene, **acenaphthylene**, anthracene, **benz[a]anthracene-[7,12]dione**, benzo[a]anthracene, benzo[a]fluoranthene, benzo[a]pyrene, **benzo[b]fluoranthene**, **benzo[e]pyrene**, benzo[ghi]perylene, **benzo[k]fluoranthene**, **chrysene**, coronene, **cyclopenta[cd]pyrene**, dibenzo[ah]anthracene, **fluoranthene**, fluorene, indeno[123-cd]pyrene, **methylchrysene**, **methylfluoranthene**, perylene, **phenanthrene**, picene, **pyrene**, **retene**, **9-fluorenone**

Table A-2: Pollutant summary statistics, Atlanta. Concentrations expressed in ng/m^3, except from OC (μg/m^3)

	Mean	St.Dev.	25%	Median	75%	% Miss
OC	3.498	2.054	2.211	3.135	4.165	23.6
n-Alkanes						
n-heneicosane	0.920	1.622	0.103	0.330	1.076	8.9
n-docosane	1.135	1.834	0.122	0.468	1.445	8.9
n-tricosane	1.880	2.262	0.447	1.050	2.380	8.9
n-tetracosane	1.580	1.784	0.498	0.975	1.988	8.9
n-pentacosane	2.041	2.314	0.696	1.296	2.585	8.9
n-hexacosane	1.466	2.044	0.463	0.888	1.663	8.9
n-heptacosane	1.701	2.340	0.608	1.065	1.998	8.9
n-octacosane	1.069	1.663	0.346	0.645	1.172	9.1
n-nonacosane	2.263	3.636	0.738	1.419	2.691	9.1
n-triacontane	0.919	1.236	0.296	0.574	1.088	9.1
n-hentriacontane	1.735	2.095	0.588	1.081	2.105	9.1
n-dotriacontane	0.499	0.662	0.130	0.274	0.589	9.1
n-tritriacontane	0.647	0.790	0.178	0.383	0.786	9.2
n-tetratriacontane	0.370	0.508	0.082	0.183	0.449	9.5
n-pentatriacontane	0.443	0.641	0.090	0.211	0.540	9.5
n-hexatriacontane	0.217	0.347	0.016	0.093	0.287	11.6
n-heptatriacontane	0.236	0.390	0.001	0.077	0.321	15.3
Iso-/Anteiso-Alkanes						
iso-nonacosane	0.113	0.193	0.031	0.064	0.125	9.2
anteiso-nonacosane	0.077	0.105	0.026	0.047	0.092	9.2
iso-triacontane	0.066	0.084	0.020	0.039	0.075	9.2
anteiso-triacontane	0.203	0.281	0.053	0.102	0.222	9.3
iso-hentriacontane	0.235	0.298	0.062	0.121	0.267	9.2
anteiso-hentriacontane	0.119	0.154	0.031	0.069	0.142	9.2
iso-dotriacontane	0.114	0.147	0.029	0.061	0.138	9.2
Cyclohexanes						
heptadecylcyclohexane	0.125	0.222	0.018	0.052	0.139	9.0
nonadecylcyclohexane	0.101	0.134	0.027	0.058	0.121	9.1
Hopanes						
$\alpha\alpha$-$\beta\alpha$-norhopane	0.052	0.129	0.013	0.027	0.049	9.2
$\alpha\alpha$-hopane	0.017	0.096	0.004	0.006	0.012	9.4
$\alpha\beta$-hopane	0.225	0.310	0.057	0.129	0.259	9.1
$\alpha\beta$-norhopane	0.355	0.512	0.080	0.196	0.405	9.1
$\alpha\beta$R-bishomohopane	0.050	0.075	0.013	0.028	0.055	9.4
$\alpha\beta$R-homohopane	0.163	0.260	0.035	0.082	0.173	9.2
$\alpha\beta$S-bishomohopane	0.067	0.093	0.017	0.038	0.076	9.4
$\alpha\beta$S-homohopane	0.178	0.273	0.037	0.090	0.196	9.2
$\beta\alpha$-hopane	0.032	0.123	0.007	0.015	0.028	9.6
22,29,30-norhopane	0.068	0.125	0.016	0.037	0.073	9.1
22,29,30-trisnorneophopane	0.078	0.113	0.021	0.046	0.085	9.1
22,29,30-trisnorphopane	0.079	0.121	0.018	0.043	0.087	9.2
22R-pentashomohopane	0.018	0.024	0.005	0.010	0.021	13.0
22R-tetrashomohopane	0.018	0.024	0.005	0.011	0.021	10.6
22R-trishomohopane	0.031	0.040	0.009	0.018	0.035	9.6
22S-pentashomohopane	0.027	0.038	0.007	0.015	0.030	13.0
22S-tretrahomohopane	0.027	0.037	0.007	0.015	0.031	10.5
22S-trishomohopane	0.051	0.092	0.013	0.027	0.056	9.6
PAH						
acenaphthylene	0.108	0.163	0.021	0.054	0.128	14.2
benzo [a] anthracene [7,12] dione	0.084	0.219	0.010	0.029	0.073	15.7
benzo [b] fluoranthene	0.169	0.493	0.016	0.043	0.126	9.6
benzo [e] pyrene	0.162	0.428	0.016	0.045	0.117	9.6
benzo [k] fluoranthene	0.156	0.402	0.017	0.044	0.129	9.4
chrysene	0.185	0.523	0.029	0.060	0.139	9.2
cyclopenta [cd] pyrene	0.222	0.692	0.002	0.017	0.135	19.1
fluoranthene	0.150	0.259	0.035	0.073	0.152	9.0
methylchrysene	0.012	0.025	0.001	0.004	0.011	21.2
methylfluoranthene	0.024	0.058	0.004	0.009	0.019	17.5
phenanthrene	0.068	0.094	0.015	0.037	0.084	8.9
pyrene	0.108	0.201	0.026	0.050	0.107	9.0
retene	0.138	0.224	0.019	0.051	0.168	9.0
9-fluorenone	0.121	0.238	0.017	0.043	0.112	9.0

Table A-3: Pollutant summary statistics, Birmingham. Concentrations expressed in ng/m^3, except from OC (μg/m^3)

	Mean	St.Dev.	25%	Median	75%	% Miss
OC	3.462	2.338	1.910	2.845	4.346	6.1
n-Alkanes						
n-heneicosane	0.879	2.116	0.024	0.270	0.853	9.1
n-docosane	1.231	2.431	0.046	0.468	1.380	9.1
n-tricosane	1.836	2.704	0.280	0.881	2.208	9.1
n-tetracosane	2.128	2.769	0.415	1.040	2.709	9.1
n-pentacosane	2.333	2.962	0.530	1.284	2.997	9.1
n-hexacosane	2.120	3.039	0.411	1.005	2.389	9.1
n-heptacosane	2.164	3.040	0.511	1.202	2.538	9.2
n-octacosane	1.312	2.019	0.329	0.723	1.421	9.3
n-nonacosane	2.538	3.938	0.669	1.517	3.060	9.4
n-triacontane	1.015	1.410	0.262	0.572	1.161	9.5
n-hentriacontane	1.840	2.218	0.582	1.207	2.288	9.4
n-dotriacontane	0.599	0.801	0.158	0.315	0.685	9.6
n-tritriacontane	0.751	0.826	0.240	0.473	0.942	9.6
n-tetratriacontane	0.524	0.690	0.120	0.272	0.607	9.8
n-pentatriacontane	0.666	0.872	0.146	0.353	0.786	10.3
n-hexatriacontane	0.320	0.507	0.030	0.125	0.390	13.3
n-heptatriacontane	0.355	0.629	0.001	0.097	0.395	17.9
Iso-/Anteiso-Alkanes						
iso-nonacosane	0.110	0.134	0.030	0.064	0.134	9.5
anteiso-nonacosane	0.098	0.140	0.025	0.052	0.108	9.4
iso-triacontane	0.067	0.096	0.018	0.037	0.073	9.5
anteiso-triacontane	0.195	0.249	0.053	0.108	0.224	9.4
iso-hentriacontane	0.209	0.249	0.053	0.119	0.255	9.5
anteiso-hentriacontane	0.142	0.159	0.042	0.091	0.180	9.5
iso-dotriacontane	0.120	0.149	0.031	0.068	0.148	9.5
Cyclohexanes						
heptadecylcyclohexane	0.133	0.240	0.014	0.053	0.145	9.4
nonadecylcyclohexane	0.122	0.167	0.024	0.064	0.143	9.6
Hopanes						
$\alpha\alpha$-$\beta\alpha$-norhopane	0.066	0.128	0.017	0.032	0.067	9.6
$\alpha\alpha$-hopane	0.018	0.065	0.005	0.009	0.016	10.3
$\alpha\beta$-hopane	0.295	0.364	0.076	0.166	0.357	9.2
$\alpha\beta$-norhopane	0.429	0.541	0.102	0.251	0.508	9.1
$\alpha\beta$R-bishomohopane	0.062	0.075	0.019	0.035	0.071	10.5
$\alpha\beta$R-homohopane	0.201	0.274	0.044	0.103	0.234	9.6
$\alpha\beta$S-bishomohopane	0.085	0.104	0.025	0.049	0.100	10.3
$\alpha\beta$S-homohopane	0.223	0.306	0.045	0.112	0.261	9.6
$\beta\alpha$-hopane	0.046	0.100	0.009	0.019	0.045	10.3
22,29,30-norhopane	0.078	0.116	0.023	0.047	0.091	9.2
22,29,30-trisnorneophopane	0.076	0.093	0.025	0.050	0.091	9.5
22,29,30-trisnorphopane	0.117	0.172	0.024	0.059	0.128	9.4
22R-pentashomohopane	0.029	0.035	0.009	0.017	0.033	18.9
22R-tetrashomohopane	0.028	0.032	0.009	0.017	0.032	15.7
22R-trishomohopane	0.043	0.049	0.014	0.026	0.051	12.0
22S-pentashomohopane	0.043	0.055	0.013	0.025	0.050	18.8
22S-tretrahomohopane	0.039	0.048	0.012	0.022	0.045	15.5
22S-trishomohopane	0.067	0.087	0.019	0.037	0.076	11.9
PAH						
acenaphthylene	0.401	0.997	0.039	0.128	0.327	13.8
benzo [a] anthracene [7,12] dione	1.661	3.989	0.044	0.224	1.036	10.7
benzo [b] fluoranthene	4.523	11.322	0.056	0.277	1.995	9.1
benzo [e] pyrene	3.273	7.816	0.042	0.252	1.682	9.1
benzo [k] fluoranthene	2.141	4.743	0.041	0.241	1.653	9.1
chrysene	3.436	7.755	0.096	0.355	2.037	9.1
cyclopenta [cd] pyrene	1.308	9.379	0.006	0.080	0.413	22.7
fluoranthene	2.236	5.410	0.089	0.257	1.446	9.1
methylchrysene	0.268	0.622	0.006	0.023	0.162	19.0
methylfluoranthene	0.722	1.919	0.013	0.048	0.389	17.9
phenanthrene	0.456	1.042	0.025	0.090	0.353	9.1
pyrene	1.804	4.330	0.061	0.184	1.106	9.1
retene	0.133	0.243	0.023	0.057	0.141	9.5
9-fluorenone	0.211	0.437	0.029	0.068	0.206	9.1

Table A-4: Pollutant summary statistics, Dallas. Concentrations expressed in ng/m^3, except from OC (μg/m^3)

	Mean	St.Dev.	25%	Median	75%	% Miss
OC	2.409	1.140	1.550	2.201	3.013	5.1
n-Alkanes						
n-heneicosane	0.924	1.334	0.134	0.425	1.002	4.9
n-docosane	1.312	1.734	0.237	0.676	1.600	4.9
n-tricosane	1.687	1.638	0.500	1.136	2.394	4.9
n-tetracosane	1.551	1.402	0.566	1.120	2.063	4.9
n-pentacosane	1.884	1.481	0.793	1.457	2.546	4.9
n-hexacosane	1.320	1.163	0.537	0.988	1.685	4.9
n-heptacosane	1.560	1.193	0.699	1.266	2.007	4.9
n-octacosane	0.919	0.907	0.349	0.642	1.139	4.9
n-nonacosane	2.171	1.911	0.924	1.702	2.922	4.9
n-triacontane	0.713	0.711	0.268	0.472	0.889	4.9
n-hentriacontane	1.700	1.848	0.561	1.151	2.305	4.9
n-dotriacontane	0.335	0.450	0.078	0.166	0.394	4.9
n-tritriacontane	0.522	0.744	0.113	0.261	0.667	4.9
n-tetratriacontane	0.166	0.301	0.020	0.057	0.162	4.9
n-pentatriacontane	0.209	0.406	0.018	0.068	0.198	4.9
n-hexatriacontane	0.079	0.177	0.001	0.004	0.078	4.9
n-heptatriacontane	0.055	0.149	0.001	0.001	0.035	4.9
Iso-/Anteiso-Alkanes						
iso-nonacosane	0.088	0.123	0.021	0.044	0.099	4.9
anteiso-nonacosane	0.060	0.097	0.012	0.026	0.067	4.9
iso-triacontane	0.043	0.059	0.010	0.022	0.050	4.9
anteiso-triacontane	0.143	0.214	0.028	0.066	0.162	4.9
iso-hentriacontane	0.117	0.165	0.026	0.057	0.136	4.9
anteiso-hentriacontane	0.069	0.108	0.011	0.030	0.078	4.9
iso-dotriacontane	0.054	0.082	0.010	0.025	0.058	4.9
Cyclohexanes						
heptadecylcyclohexane	0.131	0.184	0.023	0.064	0.156	4.9
nonadecylcyclohexane	0.093	0.108	0.031	0.060	0.105	4.9
Hopanes						
$\alpha\alpha$-$\beta\alpha$-norhopane	0.030	0.044	0.011	0.017	0.029	4.9
$\alpha\alpha$-hopane	0.006	0.008	0.002	0.004	0.007	4.9
$\alpha\beta$-hopane	0.169	0.208	0.064	0.102	0.172	4.9
$\alpha\beta$-norhopane	0.274	0.320	0.116	0.176	0.282	4.9
$\alpha\beta$R-bishomohopane	0.025	0.035	0.007	0.013	0.026	4.9
$\alpha\beta$R-homohopane	0.103	0.141	0.032	0.055	0.108	4.9
$\alpha\beta$S-bishomohopane	0.036	0.049	0.011	0.019	0.038	4.9
$\alpha\beta$S-homohopane	0.122	0.161	0.041	0.066	0.127	4.9
$\beta\alpha$-hopane	0.018	0.023	0.007	0.010	0.018	4.9
22,29,30-norhopane	0.057	0.061	0.027	0.039	0.059	4.9
22,29,30-trisnorneophopane	0.066	0.074	0.024	0.043	0.079	4.9
22,29,30-trisnorphopane	0.066	0.082	0.021	0.039	0.076	4.9
22R-pentashomohopane	0.007	0.012	0.001	0.003	0.008	4.9
22R-tetrashomohopane	0.009	0.013	0.002	0.004	0.009	4.9
22R-trishomohopane	0.015	0.020	0.004	0.008	0.016	4.9
22S-pentashomohopane	0.012	0.020	0.002	0.006	0.014	4.9
22S-tretrahomohopane	0.013	0.019	0.003	0.007	0.014	4.9
22S-trishomohopane	0.023	0.032	0.007	0.012	0.025	4.9
PAH						
acenaphthylene	0.016	0.016	0.006	0.010	0.020	4.9
benzo [a] anthracene [7,12] dione	0.038	0.072	0.001	0.013	0.041	4.9
benzo [b] fluoranthene	0.067	0.145	0.004	0.015	0.066	4.9
benzo [e] pyrene	0.079	0.168	0.006	0.017	0.075	4.9
benzo [k] fluoranthene	0.059	0.137	0.004	0.012	0.051	4.9
chrysene	0.097	0.163	0.021	0.040	0.107	4.9
cyclopenta [cd] pyrene	0.005	0.013	0.001	0.001	0.004	4.9
fluoranthene	0.111	0.123	0.047	0.074	0.127	4.9
methylchrysene	0.004	0.008	0.001	0.001	0.003	4.9
methylfluoranthene	0.009	0.017	0.002	0.004	0.010	4.9
phenanthrene	0.053	0.062	0.023	0.038	0.063	4.9
pyrene	0.064	0.093	0.022	0.038	0.067	4.9
retene	0.034	0.059	0.007	0.013	0.035	4.9
9-fluorenone	0.101	0.166	0.020	0.038	0.102	4.9

Table A-5: n-Alkane factor analysis results, by city. Correlations < 0.40 are not shown for better identification of the factor loadings.

n-Alkanes	Atlanta			Birmingham			Dallas		
	Plants	Tire Wear	Vehicles	Plants	Tire Wear	Vehicles	Plants	Tire Wear	Vehicles
n-heneicosane (C_{21})			0.93			0.93			0.64
n-docosane (C_{22})			0.94			0.94			0.71
n-tricosane (C_{23})			0.75	0.53		0.74			0.86
n-tetracosane (C_{24})	0.54		0.62	0.73		0.45			0.89
n-pentacosane (C_{25})	0.79			0.85					0.88
n-hexacosane (C_{26})	0.85			0.82				0.49	0.72
n-heptacosane (C_{27})	0.94			0.95			0.57		0.67
n-octacosane (C_{28})	0.93			0.93			0.50	0.58	0.56
n-nonacosane (C_{29})	0.95			0.92			0.87		
n-triacontane (C_{30})	0.88			0.84			0.51	0.66	0.47
n-hentriacontane (C_{31})	0.80	0.46		0.72	0.41		0.89		
n-dotriacontane (C_{32})	0.68	0.64		0.60	0.64		0.49	0.75	
n-tritriacontane (C_{33})	0.55	0.77		0.51	0.73		0.73	0.60	
n-tetratriacontane (C_{34})		0.92			0.89			0.84	0.42
n-pentatriacontane (C_{35})		0.93			0.93			0.82	
n-hexatriacontane (C_{36})		0.83			0.84			0.86	
n-heptatriacontane (C_{37})		0.76			0.81			0.81	

Table A-6: Cause-specific daily emergency hospital admissions, by city.

City	Quantiles			
	25%	50%	75%	Max
**Total Cardiovascular Admissions**				
Atlanta, GA	20	24	28	47
Birmingham, AL	6	9	11	21
Dallas, TX	15	19	22	41
**Congestive Heart Failure**				
Atlanta, GA	6	8	11	23
Birmingham, AL	1	2	4	9
Dallas, TX	4	6	8	16
**Ischemic Heart Disease**				
Atlanta, GA	5	7	9	20
Birmingham, AL	2	3	4	11
Dallas, TX	3	5	7	15
**Myocardial Infarction**				
Atlanta, GA	2	4	5	12
Birmingham, AL	1	1	2	6
Dallas, TX	2	3	4	10
**Total Respiratory Admissions**				
Atlanta, GA	14	18	22	43
Birmingham, AL	4	6	9	21
Dallas, TX	11	14	18	39
**COPD**				
Atlanta, GA	2	4	5	14
Birmingham, AL	0	1	2	7
Dallas, TX	2	3	4	11
**Pneumonia**				
Atlanta, GA	4	6	9	20
Birmingham, AL	1	2	4	11
Dallas, TX	4	5	8	21

Table A-7: Percent change (%) in total CVD and respiratory hospital admissions per 10 μg/m^3 of $PM_{2.5}$ and 1 μg/m^3 of OC and EC increase for 2- and 7-d averaged exposures.

	2-d exposures			7-d exposures		
	%Change	2.5%	97.5%	%Change	2.5%	97.5%
Cardiovascular Admissions						
$PM_{2.5}$	0.81	-0.55	2.20	0.07	-1.52	1.69
OC	**1.35**	0.06	2.64	1.13	-0.34	2.62
EC	1.08	-0.02	2.19	-0.27	-1.61	1.08
Respiratory Admissions						
$PM_{2.5}$	0.22	-1.34	1.81	-0.71	-2.54	1.15
OC	0.77	-0.65	2.22	0.36	-1.27	2.02
EC	0.81	-0.40	2.04	0.57	-0.91	2.06

Table A-8: Percent change (%) in cause specific hospital admissions per IQR increase in pollutant group for 6- and 7-d averaged exposures.

	6-d exposures			7-d exposures		
	%Change	2.5%	97.5%	%Change	2.5%	97.5%
Cardiovascular Admissions						
PAHs	-0.095	-0.477	0.288	-0.176	-0.577	0.227
Hopanes	-0.186	-0.528	0.158	-0.211	-0.569	0.149
n-Alkanes	0.072	-0.314	0.459	0.083	-0.327	0.496
Cyclohexanes	**2.898**	0.369	5.491	**3.398**	0.635	6.237
Iso-/anteiso-alkanes	-0.202	-0.785	0.384	-0.125	-0.734	0.487
CHF						
PAHs	0.153	-0.369	0.677	0.076	-0.486	0.642
Hopanes	-0.266	-0.736	0.205	-0.449	-0.951	0.055
n-Alkanes	-0.181	-0.732	0.373	-0.209	-0.808	0.394
Cyclohexanes	2.017	-1.776	5.956	3.429	-0.790	7.828
Iso-/anteiso-alkanes	0.568	-0.256	1.399	0.821	-0.050	1.700
IHD						
PAHs	-0.168	-0.696	0.364	-0.123	-0.690	0.448
Hopanes	-0.081	-0.576	0.417	0.123	-0.408	0.656
n-Alkanes	0.119	-0.458	0.699	0.133	-0.497	0.767
Cyclohexanes	3.651	-0.362	7.825	2.543	-1.803	7.081
Iso-/anteiso-alkanes	-0.573	-1.432	0.293	-0.702	-1.611	0.217
MI						
PAHs	-0.509	-1.191	0.177	-0.450	-1.187	0.293
Hopanes	-0.036	-0.675	0.607	0.068	-0.622	0.764
n-Alkanes	0.359	-0.411	1.134	0.343	-0.506	1.199
Cyclohexanes	3.214	-2.097	8.813	2.969	-2.881	9.171
Iso-/anteiso-alkanes	-0.803	-1.914	0.322	-0.915	-2.099	0.282
Respiratory Admissions						
PAHs	-0.059	-0.455	0.338	-0.104	-0.523	0.315
Hopanes	-0.330	-0.691	0.032	-0.350	-0.730	0.030
n-Alkanes	0.146	-0.264	0.558	0.145	-0.293	0.585
Cyclohexanes	0.201	-2.462	2.936	0.556	-2.349	3.547
Iso-/anteiso-alkanes	**0.635**	0.015	1.258	**0.668**	0.020	1.319
COPD						
PAHs	0.233	-0.439	0.909	0.060	-0.669	0.794
Hopanes	-0.365	-0.997	0.270	-0.476	-1.157	0.211
n-Alkanes	-0.191	-0.938	0.562	-0.163	-0.984	0.665
Cyclohexanes	3.460	-1.820	9.024	4.728	-1.134	10.938
Iso-/anteiso-alkanes	0.475	-0.641	1.604	0.580	-0.611	1.786
Pneumonia						
PAHs	0.124	-0.398	0.649	0.163	-0.400	0.730
Hopanes	-0.247	-0.737	0.244	-0.280	-0.806	0.248
n-Alkanes	0.158	-0.419	0.738	0.020	-0.608	0.653
Cyclohexanes	-3.012	-6.797	0.926	-2.257	-6.452	2.126
Iso-/anteiso-alkanes	**1.073**	0.221	1.932	**1.199**	0.300	2.107

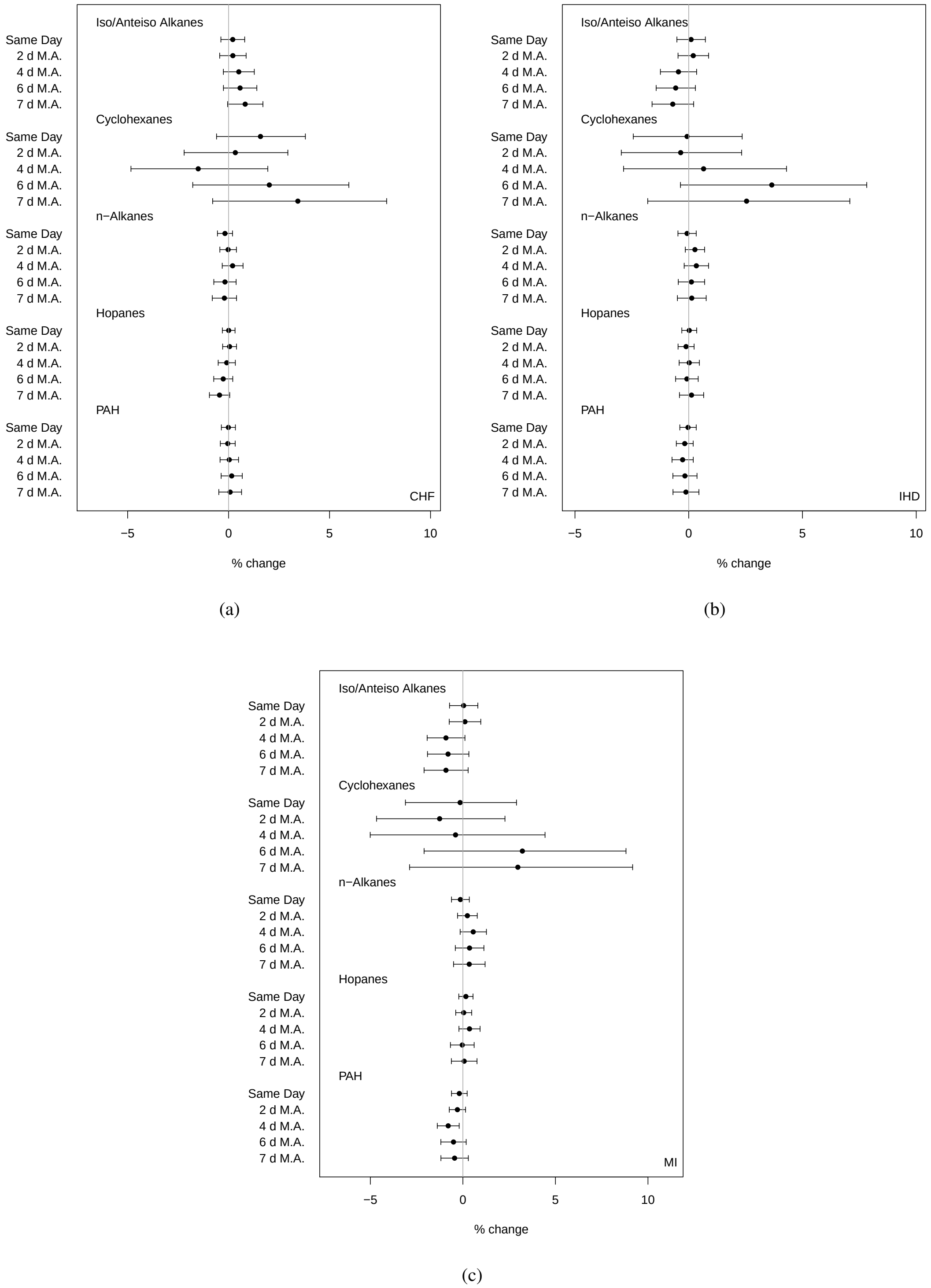

Figure A-1: Percent change in (a) CHF, (b) IHD and (c) MI hospital admissions per IQR increase in pollutant group for all exposure windows – same day exposures and 2-, 4-, 6- and 7-d moving averages (M.A.).

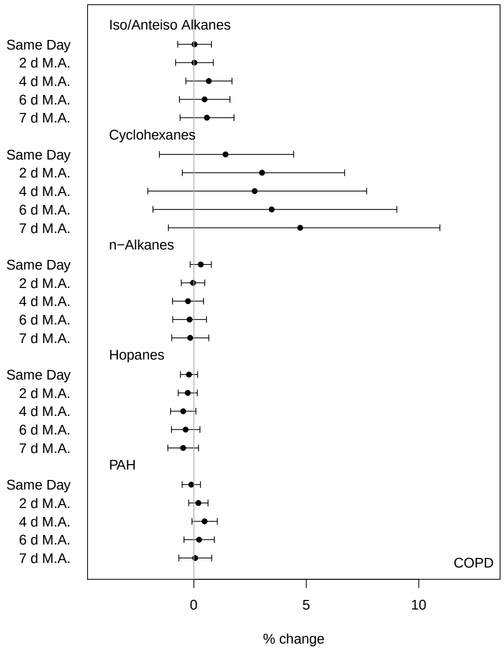

(a)

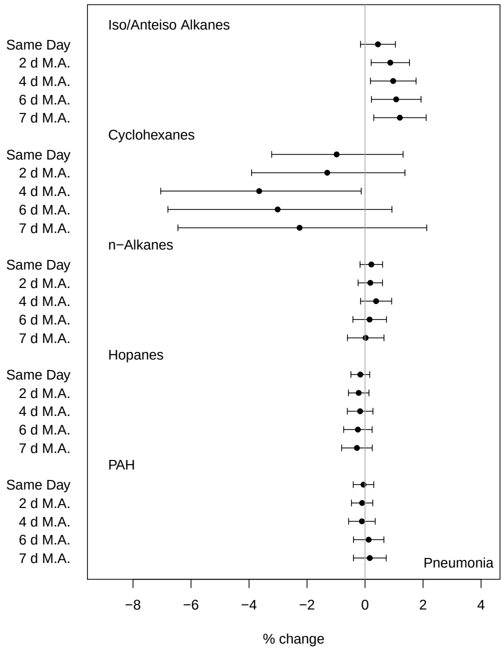

(b)

Figure A-2: Percent change in (a) COPD and (b) pneumonia hospital admissions per IQR increase in pollutant group for all exposure windows – same day exposures and 2-, 4-, 6- and 7-d moving averages (M.A.).

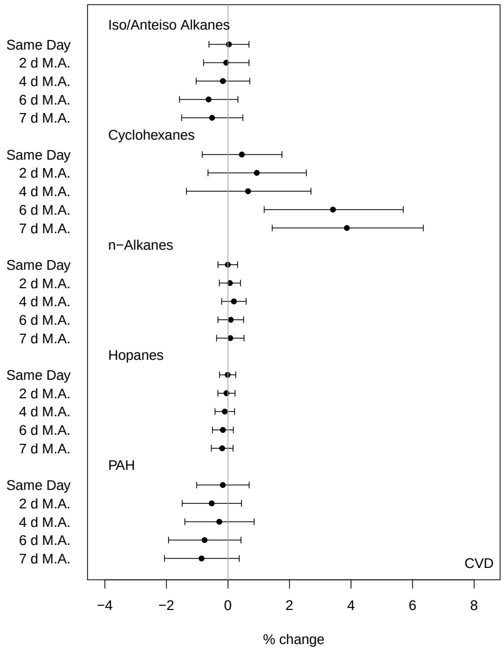

(a)

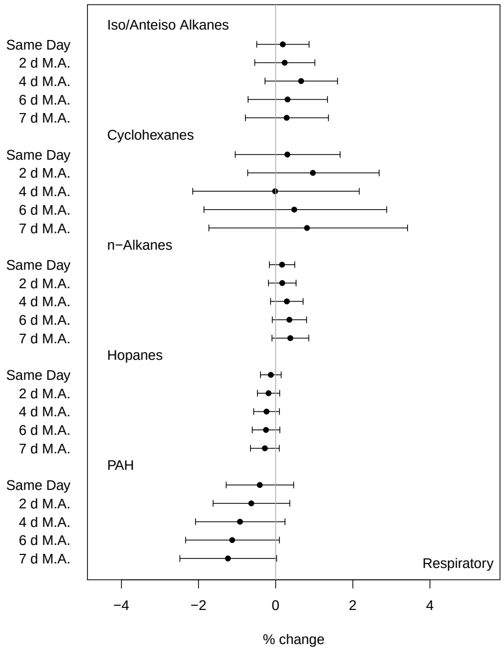

(b)

Figure A-3: Percent change in total (a) CVD and (b) respiratory hospital admissions per IQR increase in pollutant group for all exposure windows, when species with at least 75% of available observations above the LOD were included in the analysis.

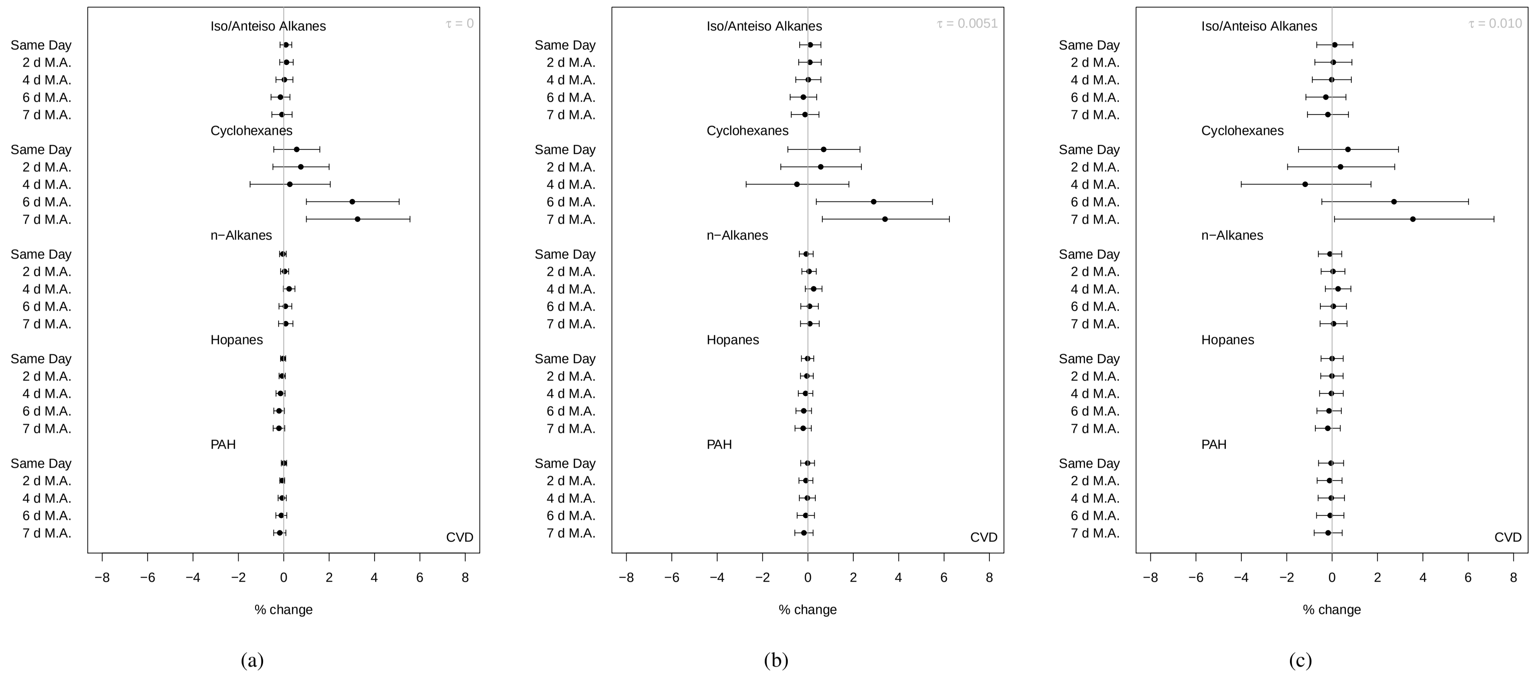

Figure A-4: Sensitivity analyses on the choice of τ^2: Percent change in total CVD admissions for (a) $\tau^2 = 0$, (b) $\tau^2 = 0.0051$ (main analysis) and (c) $\tau^2 = 0.0010$ per IQR increase in pollutant group for all exposure windows.

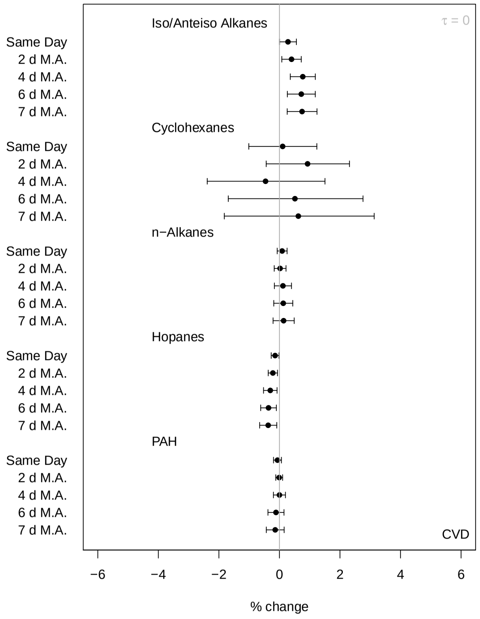

(a)

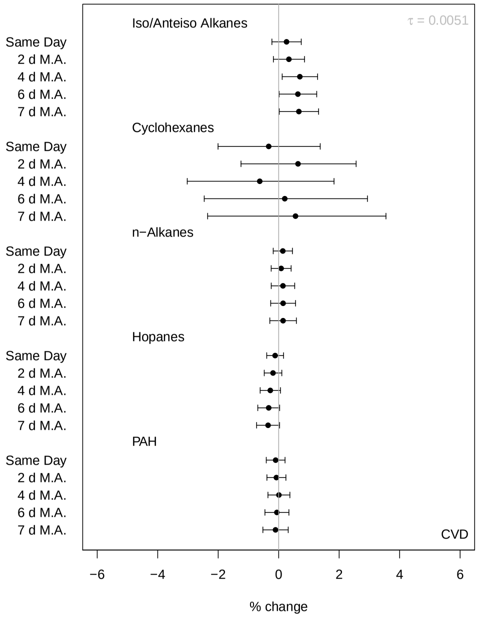

(b)

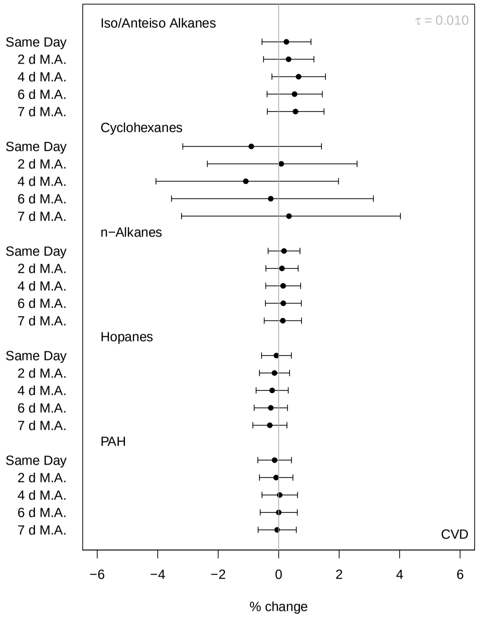

(c)

Figure A-5: Sensitivity analyses on the choice of τ^2: Percent change in total respiratory admissions for (a) $\tau^2 = 0$, (b) $\tau^2 = 0.0051$ (main analysis) and (c) $\tau^2 = 0.0010$ per IQR increase in pollutant group for all exposure windows.
